# Supplementary material for: Constraints on nonlocality in networks from no-signaling and independence
Source: Nat Commun. 2020 May 13;11:2378. doi: 10.1038/s41467-020-16137-4 (PMC7220922; doi:10.1038/s41467-020-16137-4)
Supplement: Supplementary file 1 — Supplementary information [file 41467_2020_16137_MOESM1_ESM.pdf]

Supplementary Information for: "Constraints on nonlocality in networks from no-signalling and independence"

Gisin et al.

SUPPLEMENTARY NOTE 1: NSI BOUNDS

In the hexagon configuration introduced in the main text, the probability distribution can be expressed in terms of 16 parameters as

$$\begin{aligned}
&64\,p(a,b,c,a',b',c') = \\
&1+(a+a')E_{\rm A}+(b+b')E_{\rm B}+(c+c')E_{\rm C} \\
&+(ab'+a'b)E_{\rm A}E_{\rm B}+(bc'+b'c)E_{\rm B}E_{\rm C}+(ac+a'c')E_{\rm A}E_{\rm C} \\
&+aa'E_{\rm A}^2+bb'E_{\rm B}^2+cc'E_{\rm C}^2+(ab'c+a'bc')E_{\rm A}E_{\rm B}E_{\rm C} \\
&+(ab+a'b')E_{\rm AB}+(bc+b'c')E_{\rm BC}+(ca'+c'a)E_{\rm AC} \\
&+aa'(b+b')E_{\rm A}E_{\rm AB}+aa'(c+c')E_{\rm A}E_{\rm AC} \\
&+bb'(a+a')E_{\rm B}E_{\rm AB}+bb'(c+c')E_{\rm B}E_{\rm BC} \\
&+cc'(a+a')E_{\rm C}E_{\rm AC}+cc'(b+b')E_{\rm C}E_{\rm BC} \\
&+aa'bb'E_{\rm AB}^2+bb'cc'E_{\rm BC}^2+aa'cc'E_{\rm AC}^2 \\
&+aa'(cb'+bc')E_{\rm A}F_3''+bb'(ac+a'c')E_{\rm B}F_3 \\
&+cc'(ba'+b'a)E_{\rm C}F_3' \\
&+(abc+a'b'c')F_3+(bca'+b'c'a)F_3'+(ca'b'+c'ab)F_3'' \\
&+aa'(bc+b'c')F_4+bb'(ca'+c'a)F_4'+cc'(ab+a'b')F_4'' \\
&+aa'bb'(c+c')F_5+bb'cc'(a+a')F_5'+aa'cc'(b+b')F_5'' \\
&+aa'bb'cc'F_6.
\end{aligned} \tag{1}$$

Six of these parameters, which are part of the behaviour vector $\mathcal{E}=(E_{\rm A},E_{\rm B},E_{\rm C},E_{\rm AB},E_{\rm BC},E_{\rm AC},E_{\rm ABC})$, appear in the triangle as well. We refer to them as the physical parameters. The remaining 10, from $\mathcal{F}=(F_3,F_3',F_3'',F_4,F_4',F_4'',F_5,F_5',F_5'',F_6)$, are new to the hexagon. We refer to them as free variables.

In this decomposition the E terms correspond to correlators that appear in the triangle scenario, c.f. Fig. 1(a) of the main text, whereas the free variables F_X refer to X-partite correlators in the hexagon, c.f. Fig. 1(b) of the main text. For instance, in the case of tripartite correlators (i.e. with $X=3$), the hexagon network contains three distinct tripartite correlators, which we simply refer to as F_3, F_3' and F_3''. The first one, F_3 is defined as the correlator for parties A, B, and C in the hexagon, i.e. $F_3=\sum_{a,b,c,a',b',c'}abcp(a,b,c,a',b',c')$. Since these parties are identical to A$'$, B$'$, C$'$, we also have $F_3=\sum_{a,b,c,a',b',c'}a'b'c'p(a,b,c,a',b',c')$. We notice however that this term is not identical to the tripartite correlation term $E_{\rm ABC}=\sum_{a,b,c}abcp(a,b,c)$ that can be measured in the triangle configuration (c.f. Fig 1(a) of the main text). Indeed, in the triangle configuration, parties A, B and C are connected by three sources α, β, γ, but in the hexagon configuration, these three parties are only connected by the two sources α and γ. We thus have $F_3\neq E_{\rm ABC}$. Similarly, $F_3'=\sum_{a,b,c,a',b',c'}a'bcp(a,b,c,a',b',c')$ is in general different than F_3, because this time the three parties A$'$, B, C are connected by two different sources: α and β. For the same reason we have a third tripartite correlator $F_3''=\sum_{a,b,c,a',b',c'}a'b'cp(a,b,c,a',b',c')$ in the hexagon network, and similarly for the remaining free variables.

The probabilities in the triangle configuration can be written in terms of the same variables, with an additional tripartite term $E_{\rm ABC}$, as

$$\begin{aligned}8\,p(a,b,c)=&1+aE_{\rm A}+bE_{\rm B}+cE_{\rm C}+abE_{\rm AB}\\&+bcE_{\rm BC}+acE_{\rm AC}+abcE_{\rm ABC}\end{aligned} \tag{2}$$

In this appendix, we describe the constraints that the positivity conditions $p(a,b,c,a',b',c')\geq 0$ and $p(a,b,c)\geq 0$ imply on the first six parameters.

A first general observation is that Supplementary Equation (2) is linear in its variables, but Supplementary Equation (1) is nonlinear. However, since this last expression involves no product of free variables, all nonlinearities vanish when the parameters in $\mathcal{E}$ are fixed. It is therefore always possible to test whether a behaviour given by some variables $\mathcal{E}$ is compatible with a hexagon configuration by linear programming. Concretely, this is achieved by solving the following linear program:

$$\begin{aligned}
\max_{\mathcal{F}}\quad & 1 \\
\text{s.t.}\quad & f_6(a,b,c,a',b',c',\mathcal{E},\mathcal{F})\geq 0 \qquad \forall a,b,c,a',b',c'=\pm1 \\
& f_3(a,b,c,\mathcal{E})\geq 0 \qquad \forall a,b,c=\pm1,
\end{aligned} \tag{3}$$

where $f_6(a,b,c,a',b',c',\mathcal{E},\mathcal{F})$ is the expression given by Supplementary Equation (1) and $f_3(a,b,c,\mathcal{E})$ the one given by Supplementary Equation (2). Note that this linear program involves a constant objective function because we are not trying to maximize any particular quantity, but we are rather interested in knowing whether the set of constraints admit a joint solution. If this linear program is feasible, then the behaviour given by the vector $\mathcal{E}$ is compatible with the considered constraints. Otherwise, it is not. Note that this formulation in terms of linear programming would not be possible in inflations of the triangle involving eight or more parties since in this case products of the unknown variables, such as F_3^2, appear.

In general, we are interested in more than only testing whether a behaviour is compatible with the no-signalling constraints. In particular, we would like to find NSI inequalities. For this, we start by considering simplified situations.

Uniformly random single-party marginals

Let us consider the situation in which the outputs produced by all parties are uniformly random, i.e. $E_{\rm A} = E_{\rm B} = E_{\rm C} = 0$. In this case, the expression for the probabilities (1) simplifies significantly. The number of free variables is unchanged, but all products of free variables with a physical parameter vanish. This allows us to understand the positivity constraints $p(abca'b'c') \geq 0$ as a set of linear constraints relating powers of physical parameters with some unknown variables. Inequalities involving only physical parameters can thus be obtained from this set of constraints by judiciously adding several probabilities to each other. For instance, we can write

$$\begin{aligned} 16(&p(1,1,1,-1,-1,1) + p(-1,-1,-1,1,1,-1) \\ &+ p(1,1,-1,1,1,1) + p(-1,-1,1,-1,-1,-1)) \\ =& (1 + 2E_{\rm AB} + E_{\rm AB}^2 - E_{\rm BC}^2 - E_{\rm AC}^2 + 2F_4'' + F_6 \\ &+ 1 + 2E_{\rm AB} + E_{\rm AB}^2 - E_{\rm BC}^2 - E_{\rm AC}^2 - 2F_4'' - F_6)/2 \\ =& 1 + 2E_{\rm AB} + E_{\rm AB}^2 - E_{\rm BC}^2 - E_{\rm AC}^2 \geq 0, \end{aligned} \tag{4}$$

and obtain an inequality involving no free variable in $\mathcal{F}$. More generally, all constraints on the physical variables can be obtained by performing a Fourier-Motzkin elimination on the free variables.

Performing this elimination produces 24 inequalities. After taking into account the redundancy implied by the symmetries of the Bell scenario [1], we recover the constraint (4), implying that it is tight, together with two more constraints. Altogether, these form the three following families of constraints:

$$(1 + E_{\rm AB})^2 - E_{\rm BC}^2 - E_{\rm AC}^2 \geq 0 \tag{5}$$
$$(1 + E_{\rm AB})^2 + E_{\rm BC}^2 + E_{\rm AC}^2 \geq 0 \tag{6}$$
$$1 + E_{\rm AB} + E_{\rm BC} + E_{\rm AC}^2 \geq 0. \tag{7}$$

The second inequality is a sum of squares, and therefore always true. Interestingly, the third inequality is a consequence of the first one:

$$\begin{aligned} 1+&E_{\rm AB} + E_{\rm BC} + E_{\rm AC}^2 \\ &\geq 1 + E_{\rm AB} + E_{\rm BC} - E_{\rm AC}^2 \\ &= (1 + 2E_{\rm AB} + E_{\rm AB}^2 - E_{\rm BC}^2 - E_{\rm AC}^2 \\ &\quad + 1 + 2E_{\rm BC} - E_{\rm AB}^2 + E_{\rm BC}^2 - E_{\rm AC}^2)/2 \\ &\geq 0. \end{aligned} \tag{8}$$

Therefore, the constraints due to the hexagon inflation boil down to a unique inequality : Supplementary Equation (5).

In Supplementary Figure 1, we plot the constraint imposed by this inequality in the $E_{\rm AB}$-$E_{\rm BC}$ plane for fixed values of $E_{\rm AC}$. Remarkably, most of the NSI region can be achieved by trilocal models, leaving only a small gap region (yellow area).

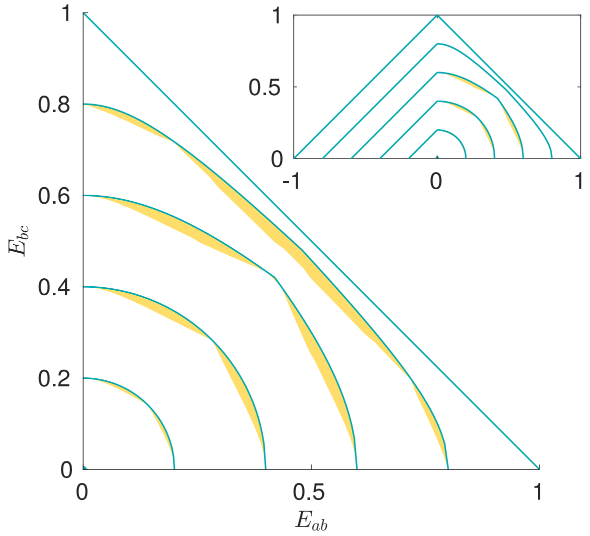

Supplementary Figure 1. Turquoise lines show the border of the NSI constraints imposed by Supplementary Inequality (5) and the positivity $p(abc) \geq 0$ for values of $E_{\rm AC}$ equal to $\{0, 0.2, 0.4, 0.6, 0.8, 1\}$ (starting from the outside). When $E_{\rm AC} = 1$, the allowed region is a single point at the origin. The inset shows the whole range of $E_{\rm AB} \in [-1, 1]$: straight lines on the left part are positivity constraints, whereas lines on the right part correspond to Supplementary Inequality (5). The curves for $E_{\rm BC} \leq 0$ can be obtained by letting Bob flip his output, hence the full figure is symmetric under a π rotation around the origin. For fixed values of $E_{\rm AC}$, the yellow area shows the "mystery" region for which we could not find a trilocal model. Note that local models can reach any part of the NSI boundary for $E_{\rm AB} \leq 0$ and $E_{\rm BC} \geq 0$.

Combining three versions of Supplementary Inequality (5), we obtain

$$\begin{aligned} &1 - 2E_{\rm AB} + E_{\rm AB}^2 - E_{\rm BC}^2 - E_{\rm AC}^2 \\ &+1 - 2E_{\rm BC} - E_{\rm AB}^2 + E_{\rm BC}^2 - E_{\rm AC}^2 \\ &+1 - 2E_{\rm AC} - E_{\rm AB}^2 - E_{\rm BC}^2 + E_{\rm AC}^2 \geq 0 \end{aligned} \tag{9}$$

which simplifies to the symmetric inequality

$$(1 + E_{\rm AB})^2 + (1 + E_{\rm BC})^2 + (1 + E_{\rm AC})^2 \leq 6. \tag{10}$$

This inequality does not detect the point $E_{\rm AB} = 1/2$, $E_{\rm BC} = -3/5$, $E_{\rm AC} = 0$, which violates Supplementary Inequality (5). The latter inequality is thus tighter.

Note that in the case where $E_{\rm AB} = E_{\rm BC} = E_{\rm AC} = E_2$, Supplementary Inequality (5) implies $E_2 \leq \sqrt{2} - 1$ whereas Supplementary Inequalities (6)-(7) are always satisfied.

We note also that the positivity constraints on the triangle impose the following condition on the bipartite marginals in presence of random marginals:

$$E_{\rm AB} + E_{\rm BC} + E_{\rm AC} \geq -1. \tag{11}$$

Since the point $E_{\rm AB} = E_{\rm BC} = E_{\rm AC} = -\sqrt{2} + 1$ satisfies inequalities in the family of Supplementary Equation (5),

but violates Supplementary Equation (11), it is not a consequence thereof.

Symmetric statistics

We now consider the special case in which the single party marginals are not all zero, but the statistics are invariant under exchange of the parties. The bipartite statistics can then be parametrized by two numbers $E_1 = E_\mathrm{A} = E_\mathrm{B} = E_\mathrm{C}$ and $E_2 = E_\mathrm{AB} = E_\mathrm{BC} = E_\mathrm{AC}$.

Under this assumption, all free parameters are still present in the decomposition (1), but the physical space is only of dimension 2. This time, however, the probabilities involve products of known with unknown variables, like $E_1 F_3$. Therefore, we cannot resort only to Fourier-Motzkin elimination to obtain all constraints that apply to the physical terms. Doing so by considering $E_1 F_3$ as a free variable to eliminate indeed would not take into account the actual value of E_1. In particular, if $E_1 = 0$, this terms would already be eliminated.

We can however resort to the linear programming formulation described earlier in Supplementary Equation (3) to describe the set of correlations in the two-dimensional space of E_1-E_2 which are compatible with the considered constraints. This gives rise to Fig. 2 presented in the main text. In particular, we verify up to numerical precision that the upper bound on E_2 as a function of E_1 of the form

$$(1 + 2E_1 + E_2)^2 \leq 2(1 + E_1)^3. \tag{12}$$

As described in Supplementary Note 2, this bound is achievable.

SUPPLEMENTARY NOTE 2: CONSTRUCTION OF TRILOCAL MODELS

In this appendix we present the explicit construction of some of the trilocal models. A trilocal model consists of (i) three distributions, for each of the shared classical variables: $\mu(\alpha)$, $\nu(\beta)$ and $\omega(\gamma)$, and (ii) three output functions (one for each party): $p_A(a|\beta,\gamma)$, $p_B(b|\alpha,\gamma)$ and $p_C(c|\alpha\beta)$. The resulting statistics is given by

$$p(a,b,c) = \int \mu(\alpha)d\alpha \int \nu(\beta)d\beta \int \omega(\gamma)d\gamma \quad p_A(a|\beta,\gamma)\, p_B(b|\alpha,\gamma)\, p_C(c|\alpha,\beta). \tag{13}$$

In a two-outcome scenario, it is sufficient to specify the output functions for outcome 1, since $p_A(a = -1|\beta,\gamma) = 1 - p_A(a = 1|\beta,\gamma)$. For ease of notation we write $p_A(a = 1|\beta,\gamma) \equiv f_a(\beta,\gamma)$. Without loss of generality, we may assume the same alphabet size, d, for all the shared variables. Hence a $d$it model can be represented by a 3-by-d matrix (with $3(d-1)$ variables) for the distributions and three d-by-d matrices, $f_a(\beta,\gamma)$, $f_b(\alpha,\gamma)$ and $f_c(\beta,\alpha)$ (each with d^2 variables) for the output functions. For example,

$$P = \left[\begin{array}{c|cc} & 1 & 2 \\ \hline \alpha & 1/2 & 1/2 \\ \beta & 1/2 & 1/2 \\ \gamma & 1/2 & 1/2 \end{array}\right], \quad f_a = f_b = f_c = \begin{bmatrix} 1 & 0 \\ 0 & 1 \end{bmatrix}, \tag{14}$$

denotes the strategies in which each source emits one bit at random and the parties output 1 whenever they receive the same bit from the two sources they are connected to.

Given a specific distribution $p(abc)$, or a specific set of marginals (e.g. E_1 and E_2), it is possible to numerically search for a trilocal model reproducing this data. Notably, for binary outcomes, it is sufficient to consider trilocal models with shared variables that have dimension $d \leq 6$ [2]. We implemented this numerical procedure, and found that for the case of low dimensions (i.e. $d = 2, 3$) the method is effective and appears to be reliable. For $d > 3$, the method can still be run, but is less reliable (i.e. the fact that the algorithm is not able to find a trilocal model does not necessarily mean that there exist none). We used this method to determine the trilocal regions in Supplementary Figs. 2 and 3 of the main text. Moreover, from the output of the numerics, we could in certain cases reconstruct analytically the trilocal models. Below we detail some of these models. Notably, these models can saturate some of the NSI constraints that we have derived, implying that the latter are tight. Finally, we also show that any point within these trilocal regions can be achieved via a trilocal model. In other words, although we characterize only the boundary of these regions, we show that any point within the boundary is also achievable (i.e. these regions do not feature any hole).

We first discuss a simple class of trilocal models, featuring only binary shared variables:

$$P = \left[\begin{array}{c|cc} & 1 & 2 \\ \hline \alpha & r & 1-r \\ \beta & q & 1-q \\ \gamma & p & 1-p \end{array}\right], \quad f_a = f_b = f_c = \begin{bmatrix} 1 & 0 \\ 0 & 0 \end{bmatrix}. \tag{15}$$

This results in the following statistics. The single-party marginals are given by

$$E_\mathrm{A} = 2pq - 1 \quad E_\mathrm{B} = 2pr - 1 \quad E_\mathrm{C} = 2qr - 1\,. \tag{16}$$

Next, the two-body marginals are

$$E_\mathrm{AB} = 1 - 2pq - 2pr + 4pqr \tag{17}$$
$$E_\mathrm{BC} = 1 - 2rp - 2rq + 4pqr \tag{18}$$
$$E_\mathrm{AC} = 1 - 2qp - 2qr + 4pqr \tag{19}$$

while the three-body correlator is

$$E_\mathrm{ABC} = -1 + 2pq + 2pr + 2qr - 4pqr\,. \tag{20}$$

This model can saturate Supplementary Inequality (12), for the case where single-party and two-body marginals

are fully symmetrical. This shows that (12) represents a tight constraint for NSI. Here, we take simply $p = q = r$ (i.e. all sources are equivalent). This leads to

$$E_1 = 2p^2 - 1 \tag{21}$$
$$E_2 = 1 - 4p^2 + 4p^3 \tag{22}$$

which corresponds to the equality condition in Eq. (12) (assuming here $p \geq 1/\sqrt{2}$, so that $E_1 \geq 0$). Note that for $p = 1/\sqrt{2}$, we get $E_1 = 0$, $E_2 = \sqrt{2} - 1$ and $E_3 = 2 - \sqrt{2}$. This model corresponds to the top-right point of the white region in Fig. 3 of the main text.

Note also that this class of trilocal models (for arbitrary values of p, q and r) saturate the conjectured NSI constraint of Eq. (8) of the main text.

Next, we present a trilocal model that achieves the $E_2 = -\frac{1}{3}$ and $E_1 = 0$:

$$P = \left[\begin{array}{c|cc} & 1 & 2 \\ \hline \alpha & 1/3 & 2/3 \\ \beta & 3/4 & 1/4 \\ \gamma & 2/3 & 1/3 \end{array}\right],$$
$$f_a = \begin{bmatrix} 1 & 0 \\ 0 & 0 \end{bmatrix}, \; f_b = \begin{bmatrix} 0 & 1/2 \\ 1/2 & 1 \end{bmatrix}, \; f_c = \begin{bmatrix} 1 & 1 \\ 0 & 1 \end{bmatrix}. \tag{23}$$

Moreover, from Fig. 2 of the main text, we see that there exist trilocal models with $E_2 = -\frac{1}{3}$ and strictly positive E_1. We find that the model that maximizes E_1 (while keeping $E_2 = -\frac{1}{3}$) is given by:

$$P = \left[\begin{array}{c|ccc} & 1 & 2 & 3 \\ \hline \alpha & x & 1-x & 0 \\ \beta & y & (1-y)/2 & (1-y)/2 \\ \gamma & 1-x & x & 0 \end{array}\right],$$
$$f_a = \begin{bmatrix} 1 & 0 & 1 \\ 0 & 0 & 0 \\ 1 & 1 & 1 \end{bmatrix}, \; f_b = \begin{bmatrix} 1 & 1 & 0 \\ 0 & 1 & 0 \\ 0 & 0 & 0 \end{bmatrix}, \; f_c = \begin{bmatrix} 0 & 1 & 0 \\ 1 & 1 & 0 \\ 0 & 0 & 0 \end{bmatrix}, \tag{24}$$

where x is the root between 0 and 1 for $3x^4 - 9x^3 + 9x^2 - 5x + 1 = 0$, and $y = \frac{1}{3(2x^2-2x+1)}$. Consequently, $E_1 = (3y^3 + y^2 + y - 1)/4 \approx 0.1753$.

Related to Fig. 3 of the main text, we now give a trilocal model for $E_1 = E_3 = 0$ and $E_2 \approx 0.3621$:

$$P = \left[\begin{array}{c|ccc} & 1 & 2 & 3 \\ \hline \alpha,\beta,\gamma & x & y & z \end{array}\right],$$
$$f_a = f_b = f_c = \begin{bmatrix} 0 & 0 & 1 \\ 0 & 0 & 0 \\ 1 & 0 & 1 \end{bmatrix}, \tag{25}$$

where $x = \frac{2}{3} - \frac{z^3}{3} - \frac{z}{2}$, $y = \frac{1}{3} + \frac{z^3}{3} - \frac{z}{2}$, and $z \approx 0.3861$ is the root between 0 and 1 for $-4z^7 + 4z^4 - 3z^3 + 8z - 3 = 0$. Consequently, $E_2 = \frac{4}{9}z^7 - \frac{8}{3}z^5 + \frac{8}{9}z^4 - z^3 + \frac{16}{3}z^2 - \frac{32}{9}z + 1 \approx 0.3621$. Note that by changing the distributions of the shared variables (but keeping the local response functions the same), one can generate the correlations indicated by the blue dots in Fig. 3 of the main text (for $0 < E_3 \leq 2 - \sqrt{2}$).

Finally, we now show that the trilocal regions shown in the various figures of the paper do not feature any hole. That is, we have characterized the boundary of these trilocal regions (in some cases by giving explicit trilocal models), and we now prove that any point inside this boundary can necessarily be achieved by a trilocal model.

The idea is to consider the following "depolarizing" protocol. Consider a distribution $p_0(abc)$ achievable via a trilocal model M, given by single-party marginals E^0_{A}, etc..., bipartite marginals E^0_{AB}, etc..., and a tripartite correlator E^0_{ABC}. Each party adds noise locally (and independently of the other parties) via the following procedure. With probability $1-\eta$ a party provides a random output, while with probability η they output according to M. Hence we obtain the continuous family of distributions characterized by $E_{\mathrm{A}} = \eta E^0_{\mathrm{A}}$ etc, $E_{\mathrm{AB}} = \eta^2 E^0_{\mathrm{AB}}$ etc and $E_{\mathrm{ABC}} = \eta^3 E^0_{\mathrm{ABC}}$. Varying η from 1 to 0 we obtain a continuous curve from $p_0(abc)$ to the uniform distribution.

For Figs. 2 and 3 of the main text and Supplementary Figure 1, we see that any point inside the trilocal region can be obtained by adding (a well chosen) amount of noise to a distribution sitting on the boundary. For Supplementary Figure 2, the situation is different, as the above depolarizing procedure takes an initial distribution on the slice outside of it.

SUPPLEMENTARY NOTE 3: COMPARISON TO FINNER INEQUALITY

We compare the NSI constraint derived here with a criterion derived in Ref. [3]. The later is based on the Finner inequality, and states that

$$p(abc) \leq \sqrt{p_A(a)p_B(b)p_C(c)} \tag{26}$$

where $p_A(a)$ represents Alice's marginal probability to observe outcome a, and similarly for $p_B(b)$ and $p_C(c)$. Importantly, it should be pointed out that the above inequality is only conjectured to hold under NSI. At the moment, it is only proven that inequality (26) holds in quantum theory, and in "boxworld" (a generalized probabilistic theory where sources can prepare arbitrary no-signalling boxes, see e.g. [4]). This does not imply that (26) holds in any generalized probabilistic theory.

Nevertheless it is interesting to compare both approaches, in particular as inequality (26) involves explicitly tripartite correlations (e.g. the term $p(abc)$), contrary to our approach which seems to be limited to single- and two-party marginals.

To perform this comparison, we use a specific set of distributions in the triangle network, discussed in [3]. These

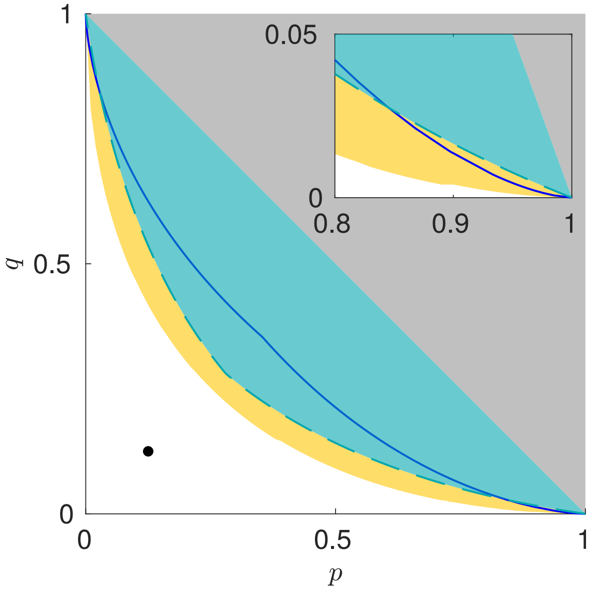

Supplementary Figure 2. Comparison of our NSI constraint (12) and the Finner inequality (26) of Ref. [3] (solid blue curve represents equality in (26)), for the set of distributions $p_{p,q}$ given in Eq. (27). Our NSI constraints (dashed turquoise curve representing equality in (12)) appears to be stronger almost everywhere, except for two small regions, around each deterministic point P_{+++} ($p = 1$) and P_{---} ($q = 1$); see inset. As in previous figures, the grey region is excluded via positivity constraints and the turquoise region via NSI constraints. The white region is achievable via trilocal models, while the yellow region is undetermined. The black point represents the uniformly random distribution, i.e. $p = q = 1/8$.

take the form

$$p_{p,q} = pP_{+++} + qP_{---} + (1-p-q)P_{\text{diff}} \tag{27}$$

where P_{abc} represents the distribution where the outputs are set to values a, b and c deterministically, and $P_{\text{diff}} = (P_{++-}+P_{+-+}+P_{-++}+P_{+--}+P_{-+-}+P_{--+})/6$. From (26) it follows that $p_{p,q}$ is not realizable in the triangle network when $q > 1 + p - 2p^{2/3}$ (and a similar constraint inverting p and q); given by the black curve in Supplementary Figure 2.

We compare this criterion to our NSI constraint (12). From Supplementary Figure 2, we see that our NSI constraint is mostly stronger than the Finner inequality (26). However, this is not the case in general, as there is a small region (around the deterministic points P_{+++} and P_{---}) where the Finner inequality is stronger.

SUPPLEMENTARY NOTE 4: PROOF OF A GENERAL NSI INEQUALITY

Here we prove the validity of the following inequality for NSI models in the triangle network:

$$\begin{aligned}&(1+|E_{\text{A}}|+|E_{\text{B}}|+E_{\text{AB}})^2\\&+(1+|E_{\text{A}}|+|E_{\text{C}}|+E_{\text{AC}})^2\\&+(1+|E_{\text{B}}|+|E_{\text{C}}|+E_{\text{BC}})^2\\&\leq 6(1+|E_{\text{A}}|)(1+|E_{\text{B}}|)(1+|E_{\text{C}}|)\,.\end{aligned} \tag{28}$$

This inequality is invariant under exchange of parties. It is also invariant under the joint relabelling of all parties' outputs. However, it is not invariant under arbitrary output relabelling. Therefore, we consider two cases:

1. If $E_{\text{A}}, E_{\text{B}}, E_{\text{C}} \geq 0$, Supplementary Equation (28) can be written

$$\begin{aligned}&(1+E_{\text{A}}+E_{\text{B}}+E_{\text{AB}})^2\\&+(1+E_{\text{A}}+E_{\text{C}}+E_{\text{AC}})^2\\&+(1+E_{\text{B}}+E_{\text{C}}+E_{\text{BC}})^2\\&\leq 6(1+E_{\text{A}})(1+E_{\text{B}})(1+E_{\text{C}})\,.\end{aligned} \tag{29}$$

   If $E_{\text{A}}, E_{\text{B}}, E_{\text{C}} \leq 0$, flipping all outcomes brings us back to the same condition.

2. In all other cases we can always exchange parties and outcomes (jointly) to reach the case $E_{\text{A}}, E_{\text{C}} \geq 0$, $E_{\text{B}} \leq 0$. In this case, Supplementary Equation (28) reduces to

$$\begin{aligned}&(1+E_{\text{A}}-E_{\text{B}}+E_{\text{AB}})^2\\&+(1+E_{\text{A}}+E_{\text{C}}+E_{\text{AC}})^2\\&+(1-E_{\text{B}}+E_{\text{C}}+E_{\text{BC}})^2\\&\leq 6(1+E_{\text{A}})(1-E_{\text{B}})(1+E_{\text{C}})\,.\end{aligned} \tag{30}$$

It is thus sufficient to show the validity of the two inequalities (29) and (30) in their respective context.

Before focusing on these cases, we make some general observations. Since probabilities are positive, the following sum of probabilities also is:

$$\begin{aligned}&p(-1,1,-1,1,1,1)+p(1,-1,1,-1,-1,-1)\\&+p(-1,-1,1,-1,1,1)+p(1,1,-1,1,-1,-1)\geq 0.\end{aligned} \tag{31}$$

Using Supplementary Equation (1), this condition can be rewritten as the following inequality:

$$1 - 2E_{\text{AC}} + 2E_{\text{B}}F_3 \geq E_{\text{AB}}^2 - E_{\text{AC}}^2 + E_{\text{BC}}^2. \tag{32}$$

Similarly, $\sum_{a',b',c'} p(1,-1,1,a',b',c') \geq 0$ implies

$$I_2 = 1 + E_{\text{A}} - E_{\text{B}} + E_{\text{C}} + E_{\text{A}}E_{\text{C}} - E_{\text{AB}} - E_{\text{BC}} - F_3 \geq 0, \tag{33}$$

and $\sum_{a',b',c'} p(-1,1,-1,a',b',c') \geq 0$ implies

$$I_2' = 1 - E_\mathrm{A} + E_\mathrm{B} - E_\mathrm{C} + E_\mathrm{A}E_\mathrm{C} - E_\mathrm{AB} - E_\mathrm{BC} + F_3 \geq 0, \quad (34)$$

Case 1 ($E_\mathrm{A}, E_\mathrm{B}, E_\mathrm{C} \geq 0$): Since $E_\mathrm{B} \geq 0$, the product $E_\mathrm{B} I_2$ (33) is positive. After rearrangement, we obtain

$$\begin{aligned} &2(1+E_\mathrm{A})(1+E_\mathrm{B})(1+E_\mathrm{C}) - (1+E_\mathrm{A}+E_\mathrm{C}+E_\mathrm{AC})^2 \\ &\geq -E_\mathrm{A}^2 + 2E_\mathrm{B}^2 - E_\mathrm{C}^2 + 2E_\mathrm{B}(E_\mathrm{AB}+E_\mathrm{BC}) \\ &\quad - 2E_\mathrm{AC}(E_\mathrm{A}+E_\mathrm{C}) - E_\mathrm{AC}^2 + 1 - 2E_\mathrm{AC} + 2E_\mathrm{B}F_3. \end{aligned} \quad (35)$$

Recognizing the last three terms from (32), we use this inequality to eliminate the term containing the free variable F_3 and get

$$\begin{aligned} &2(1+E_\mathrm{A})(1+E_\mathrm{B})(1+E_\mathrm{C}) - (1+E_\mathrm{A}+E_\mathrm{C}+E_\mathrm{AC})^2 \\ &\geq -E_\mathrm{A}^2 + 2E_\mathrm{B}^2 - E_\mathrm{C}^2 + 2E_\mathrm{B}(E_\mathrm{AB}+E_\mathrm{BC}) \\ &\quad - 2E_\mathrm{AC}(E_\mathrm{A}+E_\mathrm{C}) + E_\mathrm{AB}^2 - 2E_\mathrm{AC}^2 + E_\mathrm{BC}^2. \end{aligned} \quad (36)$$

Since E_C is positive, this inequality remains valid if we cyclically permute the parties to let C play the role of B, yielding

$$\begin{aligned} &2(1+E_\mathrm{A})(1+E_\mathrm{B})(1+E_\mathrm{C}) - (1+E_\mathrm{A}+E_\mathrm{B}+E_\mathrm{AB})^2 \\ &\geq -E_\mathrm{A}^2 - E_\mathrm{B}^2 + 2E_\mathrm{C}^2 + 2E_\mathrm{C}(E_\mathrm{AC}+E_\mathrm{BC}) \\ &\quad - 2E_\mathrm{AB}(E_\mathrm{A}+E_\mathrm{B}) - 2E_\mathrm{AB}^2 + E_\mathrm{AC}^2 + E_\mathrm{BC}^2. \end{aligned} \quad (37)$$

Similarly, since $E_\mathrm{A} \geq 0$ we can also write

$$\begin{aligned} &2(1+E_\mathrm{A})(1+E_\mathrm{B})(1+E_\mathrm{C}) - (1+E_\mathrm{B}+E_\mathrm{C}+E_\mathrm{BC})^2 \\ &\geq 2E_\mathrm{A}^2 - E_\mathrm{B}^2 - E_\mathrm{C}^2 + 2E_\mathrm{A}(E_\mathrm{AB}+E_\mathrm{AC}) \\ &\quad - 2E_\mathrm{BC}(E_\mathrm{B}+E_\mathrm{C}) + E_\mathrm{AB}^2 + E_\mathrm{AC}^2 - 2E_\mathrm{BC}^2. \end{aligned} \quad (38)$$

Summing up the three Supplementary Equations (36), (37), (38), all terms on the right-hand side cancel out, leaving us with:

$$\begin{aligned} &6(1+E_\mathrm{A})(1+E_\mathrm{B})(1+E_\mathrm{C}) - (1+E_\mathrm{A}+E_\mathrm{C}+E_\mathrm{AC})^2 \\ &- (1+E_\mathrm{A}+E_\mathrm{B}+E_\mathrm{AB})^2 - (1+E_\mathrm{B}+E_\mathrm{C}+E_\mathrm{BC})^2 \geq 0. \end{aligned} \quad (39)$$

This inequality is identical to (29), which concludes the proof under the $E_\mathrm{A} \geq 0, E_\mathrm{B} \geq 0, E_\mathrm{C} \geq 0$ assumption.

Case 2 ($E_\mathrm{A}, E_\mathrm{C} \geq 0$, $E_\mathrm{B} \leq 0$): This time, E_B is negative, so we cannot use Supplementary Equation (36). However, we still have $E_\mathrm{A} \geq 0$ and $E_\mathrm{C} \geq 0$ so Supplementary Equations (37) and (38) remain valid. For clarity, we rearrange them as

$$\begin{aligned} &2(1+E_\mathrm{A})(1-E_\mathrm{B})(1+E_\mathrm{C}) - (1+E_\mathrm{A}-E_\mathrm{B}+E_\mathrm{AB})^2 \\ &\geq -E_\mathrm{A}^2 - E_\mathrm{B}^2 + 2E_\mathrm{C}^2 - 2E_\mathrm{AB}^2 + E_\mathrm{BC}^2 + E_\mathrm{AC}^2 \\ &\quad + 2E_\mathrm{B}E_\mathrm{AB} - 2E_\mathrm{A}E_\mathrm{AB} + 2E_\mathrm{C}(E_\mathrm{AC}+E_\mathrm{BC}) \\ &\quad - 4E_\mathrm{B}(E_\mathrm{C}+E_\mathrm{A}E_\mathrm{C}) \end{aligned} \quad (40)$$

and

$$\begin{aligned} &2(1+E_\mathrm{A})(1-E_\mathrm{B})(1+E_\mathrm{C}) - (1-E_\mathrm{B}+E_\mathrm{C}+E_\mathrm{BC})^2 \\ &\geq 2E_\mathrm{A}^2 - E_\mathrm{B}^2 - E_\mathrm{C}^2 + E_\mathrm{AB}^2 - 2E_\mathrm{BC}^2 + E_\mathrm{AC}^2 \\ &\quad + 2E_\mathrm{B}E_\mathrm{BC} - 2E_\mathrm{C}E_\mathrm{BC} + 2E_\mathrm{A}(E_\mathrm{AB}+E_\mathrm{AC}) \\ &\quad - 4E_\mathrm{B}(E_\mathrm{A}+E_\mathrm{A}E_\mathrm{C}) \end{aligned} \quad (41)$$

Considering now the positive expression I_2' in Supplementary Equation (34), we multiply it with $-E_\mathrm{B}$, which is positive. After rearrangement, this gives

$$\begin{aligned} &2(1+E_\mathrm{A})(1-E_\mathrm{B})(1+E_\mathrm{C}) - (1+E_\mathrm{A}+E_\mathrm{C}+E_\mathrm{AC})^2 \\ &\geq -E_\mathrm{A}^2 + 2E_\mathrm{B}^2 - E_\mathrm{C}^2 - 2E_\mathrm{B}(E_\mathrm{AB}+E_\mathrm{BC}) \\ &\quad - 2E_\mathrm{AC}(E_\mathrm{A}+E_\mathrm{C}) - 4E_\mathrm{B}(E_\mathrm{A}+E_\mathrm{C}) - E_\mathrm{AC}^2 \\ &\quad + 1 - 2E_\mathrm{AC} + 2E_\mathrm{B}F_3 \end{aligned} \quad (42)$$

Recognizing again the last three terms from Supplementary Equation (32), we obtain

$$\begin{aligned} &2(1+E_\mathrm{A})(1-E_\mathrm{B})(1+E_\mathrm{C}) - (1+E_\mathrm{A}+E_\mathrm{C}+E_\mathrm{AC})^2 \\ &\geq -E_\mathrm{A}^2 + 2E_\mathrm{B}^2 - E_\mathrm{C}^2 - 2E_\mathrm{B}(E_\mathrm{AB}+E_\mathrm{BC}) \\ &\quad - 2E_\mathrm{AC}(E_\mathrm{A}+E_\mathrm{C}) - 4E_\mathrm{B}(E_\mathrm{A}+E_\mathrm{C}) \\ &\quad + E_\mathrm{AB}^2 + E_\mathrm{BC}^2 - 2E_\mathrm{AC}^2 \end{aligned} \quad (43)$$

Summing Supplementary Equations (40), (41) and (43), we obtain

$$\begin{aligned} &6(1+E_\mathrm{A})(1-E_\mathrm{B})(1+E_\mathrm{C}) - (1+E_\mathrm{A}+E_\mathrm{C}+E_\mathrm{AC})^2 \\ &- (1+E_\mathrm{A}-E_\mathrm{B}+E_\mathrm{AB})^2 - (1-E_\mathrm{B}+E_\mathrm{C}+E_\mathrm{BC})^2 \\ &\geq -8E_\mathrm{B}(E_\mathrm{A}+E_\mathrm{C}+E_\mathrm{A}E_\mathrm{C}) \geq 0, \end{aligned} \quad (44)$$

where the positivity follows from the negativity of E_B and the positivity of E_A and E_C. This proves (30) under the $E_\mathrm{A} \geq 0, E_\mathrm{B} \leq 0, E_\mathrm{C} \geq 0$ assumptions, which concludes the proof of Supplementary Equation (28).

SUPPLEMENTARY REFERENCES

[1] Rosset, D., Bancal, J.-D. & Gisin, N. Classifying 50 years of Bell inequalities. *Journal of Physics A: Mathematical and Theoretical* **47**, 424022 (2014).

[2] Rosset, D., Gisin, N. & Wolfe, E. Universal bound on the cardinality of local hidden variables in networks. *Quantum Information & Computation* **18**, 910–926 (2018).

[3] Renou, M.-O. *et al.* Limits on correlations in networks for quantum and no-signaling resources. *Physical Review Letters* **123**, 070403 (2019).

[4] Barrett, J. Information processing in generalized probabilistic theories. *Physical Review A* **75**, 032304 (2007).
